# Conceptual and empirical item hierarchies of person-centred outpatient care

**DOI:** 10.1016/j.heliyon.2024.e41163

**Published:** 2024-12-12

**Authors:** Fredrik Gasser, Albert Westergren, Sidona-Valentina Bala, Joakim Ekstrand, Peter Hagell

**Affiliations:** a The PRO-CARE Group, Faculty of Health Sciences, Kristianstad University, Kristianstad, Sweden; b The Research Platform for Collaboration for Health, Faculty of Health Sciences, Kristianstad University, Kristianstad, Sweden; c Department of Health Sciences, Faculty of Medicine, Lund University, Lund, Sweden; d Department of Medicine, Section of Rheumatology, Helsingborg Central Hospital, Helsingborg, Sweden

## Abstract

**Introduction:**

The Person-Centred Care instrument for outpatient care (PCCoc) is a 36-item patient-reported experience measure with 4 ordered response categories, that aims to capture the degree of perceived person-centred care (PCC) from a patient perspective among persons with long-term conditions. The PCCoc is based on a framework that conceptualises outpatient PCC from lower to higher levels of perceived PCC, from personalisation via shared decision-making to empowerment, where 35 of the PCCoc items are a part of the framework's hierarchy.

**Aim:**

To investigate to what extent empirical item responses are consistent with the hierarchical PCCoc conceptual framework among persons with long-term conditions in outpatient care.

**Methods:**

PCCoc data (323 responses) from persons with long-term psychiatric, cardiological, rheumatological or neurological conditions were analysed. The Rasch measurement model (RMM) was used to evaluate model fit and the empirical item ordering. Correspondence between the empirical and conceptually expected item hierarchies was assessed graphically and using the polyserial correlation between RMM derived item locations and their a-priori expected rank order.

**Result:**

Two items showed clear misfit to the RMM. The polyserial correlation between empirical item locations and the expected rank order using all 35 PCCoc items was 0.64; after removing the 2 misfitting items it was 0.71. In addition, subtests (i.e., testlets consisting of a combination of items belonging to the respective hierarchical domains) were created to account for any local dependency. Testlet locations on the hierarchical continuum indicated good correspondence between empirical data and the conceptual hierarchy when based on 35 as well as 33 items. Both testlet analyses had a polyserial correlation of 0.99 between testlet locations and the expected rank order.

**Conclusion:**

The observed correspondence between empirical data and the conceptual framework indicates that the PCCoc reflects the underlying framework, and therefore can be a valuable instrument to support targeted PCC-promoting interventions.

## 1 Introduction

An essential part of a Person-centred care (PCC) is the relationship between the healthcare professional (HCP) and the person in need of care, a relationship that promotes mutual recognition of important goals and strengthens the ability to achieve them [1]. A more person-centred care would be beneficial for persons with long-term conditions (LTCs) [2,3], with potential benefits such as improved satisfaction with care; enhanced relationships with HCPs; improved ability to make decisions that foster independence; shared decision-making with HCPs that increase participation in care planning; improved capacity to self-manage and control LTC [3]. However, it has been shown that persons with more complex care needs often receive a less person-centred outpatient care [4] and several barriers to implement PCC have been identified [5].

This prompts a need for valid and reliable instruments for monitoring, developing, and evaluating PCC for persons with LTC, which appear scarce in outpatient care [6,7]. The development of robust instruments, however, is challenging, since PCC is a latent variable, i.e., not directly observable. Therefore, to generate meaningful measurement of the latent variable an a-priori understanding of the expected order of the items that form the instrument is needed [8,9]. With this comes a requirement that the item order from less to more on the latent variable reflects something important and meaningful about the underlying construct, which calls for a clearly defined conceptual framework [10,11].

**Fig. 1:**
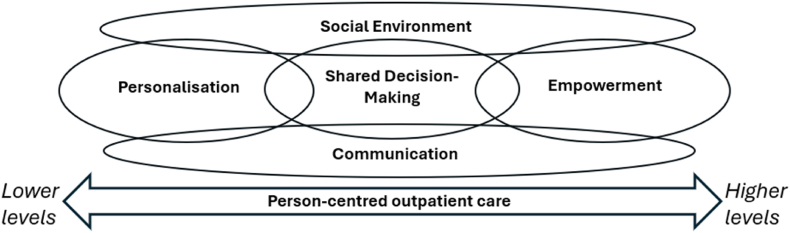
The Conceptual framework for person-centred outpatient care (Bala et al., 2018b).

The Person-Centred Care instrument for outpatient care (PCCoc) is a 36-item patient reported experience measure (PREM) with 4 ordered response categories (Totally disagree; Disagree; Agree; Completely agree), that aims to capture the degree of perceived PCC from a patient perspective among persons with LTCs in outpatient care. Evaluations of the PCCoc showed user-friendliness and relevance for persons with LTCs in different outpatient settings [12,13]. The instrument is based on a conceptual framework for person-centred outpatient care developed by Bala et al. [14] in a nurse-led rheumatology context, that conceptualises PCC on a latent continuum from lower to higher levels of perceived person-centredness. The conceptual framework was developed through qualitative interviews with persons in rheumatological outpatient care and theoretical considerations. The framework conceptualises a latent hierarchical continuum ranging from personalisation (lower levels of PCC) via shared decision-making to empowerment (higher levels of PCC), where adjacent domains are expected to overlap. Lower levels represent aspects of PCC that are relatively easy to achieve, whereas higher levels are more difficult to achieve. Two additional domains, social environment and communication, are not considered part of the hierarchy but integrated throughout the continuum (Fig. 1). Personalisation involves identifying and acknowledging the needs and abilities of the unique person. Shared decision-making involves collaboration between health professionals and the person in need of care, where information is shared and health professionals support decision-making. Empowerment involves enabling the person's own ability to cope with and control their situation. Social environment involves both how a person is approached as well as the physical environment, and communication involves the co-creation of a dialogue that establishes a relationship and mutual understanding [14].

Based on the conceptual framework, items were primarily identified from qualitative interviews, with the purpose to reflect the hierarchical order from lower to higher levels of perceived person-centred outpatient care in the conceptual framework. This resulted in a 24-item PREM, the PCCoc/rheum, intended for use in nurse-led rheumatological outpatient care [14]. Subsequently, Bala et al. [15] explored the correspondence between the a-priori hypothesized item hierarchy and the conceptual framework by using the Rasch measurement model (RMM) [16,17]. This can be done since the RMM locates items along a linear continuum from lower to higher levels of the measured construct. Items representing lower levels on the continuum are less demanding and associated with a higher probability to be achieved than those located higher on the continuum. Correspondence between the empirical and theoretically expected item hierarchies can thereafter be assessed using the polyserial correlation between linear RMM derived item locations and their expected rank order. The initial correlation between the empirical and conceptually expected PCCoc/rheum item hierarchies was 0.22, which increased to 0.83 after three items were deleted based on theoretical and statistical considerations [15]. Thereafter the PCCoc/rheum has been further modified for generic use (i.e., irrespective of the health professional responsible for the care or the outpatient unit providing care), including the addition of 12 new items. This generic version is called the PCCoc [12] and is, to the best of our knowledge, the first PREM for persons with long-term conditions in outpatient settings based on a conceptual framework that defines PCC from lower to higher levels. Thirty-five of the 36 PCCoc items are part of the hierarchical continuum from lower to higher levels of PCC, and one item exclusively represents social environment.

Although the PCCoc has been found to be user-friendly and relevant for persons with LTCs in outpatient care [12,13], it is unknown to what extent the generic PCCoc corresponds with the underpinning hierarchical conceptual framework when applied in various outpatient settings. There is thus a need to investigate whether empirical PCCoc item response data are consistent with the conceptual item hierarchy, as an important step in the development of a PREM targeting perceived person-centred outpatient care for persons with LTCs based on a clearly defined framework for person-centred outpatient care.

### 1.1 Aim

To investigate to what extent empirical item responses are consistent with the hierarchical PCCoc conceptual framework among persons with long-term conditions in outpatient care.

## 2 Methods

### 2.1 Sample and data collection

**Fig. 2:**
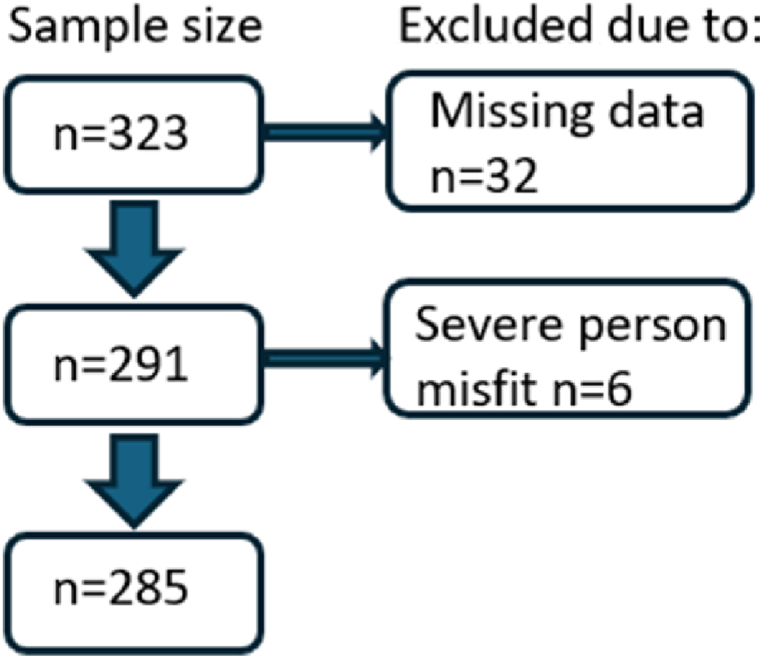
Sample size. n denotes available responses from the two timepoints. Only complete data were included, and observations with severe misfit (fit residuals >4) were excluded.

Participants were recruited at two independent occasions by HCPs at specialist outpatient clinics providing care for persons with major LTCs (psychiatry, cardiology, rheumatology, and neurology) during 2021–2022 [12]. The outpatient clinics were included to cover a variety of somatic and psychiatric LTCs. Swedish speaking persons with a LTC and who had had at least three contacts with their respective outpatient clinic during the past year were eligible for inclusion. The HCPs provided oral and written information about the study and collected written informed consent. Participants received a background questionnaire and two copies of the PCCoc, which they were instructed to complete independently approximately 14 days apart. This resulted in 323 responses from 179 persons with long-term psychiatric, cardiological, rheumatological or neurological conditions (Fig. 2). Differences in item functioning between the two timepoints was examined at the outset of the analyses, were an absence of differential item functioning by time was taken as support for merging data from the two timepoints, as it can gain an increased precision of estimates [18].

The distribution of participants was evenly spread across the different outpatient clinics. The mean (SD) age for the whole sample was 55.8 (16.9) years; 50.3 % were women; most participants (64.2 %) were married; 38.8 % reported poor to fair health, 28 % good health and 33.2 % very good to excellent health. A more detailed description of the sample and data collection can be found in Gasser et al. [12].

### 2.2 Data analyses

PCCoc item responses were analysed according to Rasch measurement theory (RMT) [16] using the unrestricted polytomous (“partial credit”) RMM [17]. Analyses were conducted with complete data (n = 291) (Fig. 2).

In the RMM the probability of a certain item response is governed by the person's location on the latent variable (here, the perceived degree of PCC) and what degree of PCC the item represents. The location of persons and items are estimated separately on a common linear logit (log-odds units) scale, ranging from -∞ to +∞ and with mean item location set at zero [17]. Since invariance is a consequence of the model, it also follows if data fit the RMM [19]. In this study, the following analyses were conducted:

*Targeting* refers to the extent to which the items represent the levels of perceived PCC reported by the sample and, vice versa, i.e., the extent to which the sample distribution covers the levels of perceived PCC represented by the items. Mistargeting can negatively affect measurement precision [20].

*Reliability* was estimated using the person separation index (PSI), conceptually analogous to Cronbach's alpha, but based on estimates derived from the RMM instead of raw scores [17]. PSI can be used to determine the number of strata (i.e., the number of statistically distinct groups of persons that can be distinguished by the instrument) [21].

*Fit between the data and the model* was examined using several related approaches: Item characteristic curves (ICC) provide a graphic depiction of the relationship between observed and expected responses. Discrepancies between the observed item response and that expected by the model are also expressed as standardized item fit residuals. The expected residual value is 0 but considered acceptable in the range of ±2.5 [17]. Item-level approximate chi-square statistics formalize the difference between observed and expected item responses, which also can be expressed as an item-trait interaction statistic for the whole instrument [17]. Chi-square associated p-values indicate whether the difference may be due to chance [19]. In addition, person fit, i.e., the residual between the expected response according to the RMM and the person's item response [17] was evaluated. Strongly deviating response patterns (i.e., high positive residuals) can affect item fit [22]. In this study, responses with a fit residual >4 were therefore omitted (Fig. 2).

*Differential Item Functioning (DIF)* is an aspect of model fit and concerns the extent to which items function invariantly across different subgroups. DIF occurs when an item lacks invariance across different groups of persons that are matched according to their locations on the trait. DIF was examined statistically using a two-way ANOVA of items residuals across levels of the latent trait (here, PCC) [17,23]. Uniform DIF occurs when subgroups of persons (e.g., men and women) systematically differ in their responses to an item across locations on the latent continuum. In non-uniform DIF, the differences between responses to an item between subgroups varies across levels of the attribute [22]. In addition to time (see above), DIF was tested for age (two subgroups based on median age, <60 and ≥ 60 years old) and sex (men and women).

*Response category functioning* assesses if ordered response categories function as intended, i.e., if they represent a continuum of increasing levels from less to more (e.g., Totally disagree < Disagree < Agree < Completely agree), as represented by the ordering of the response category thresholds. Thresholds are the locations where there are equal probabilities that a person respond in either of two adjacent categories. Disordered thresholds indicate a malfunctioning in the response categories [17,19].

*Local dependence* (LD) is a violation of the RMM, which rely on the assumption of local independence, i.e., that it is the person parameter that accounts for all the variation among responses to an item [24]. LD may reflect either response or trait dependence (i.e., multidimensionality) [25]. Response dependence implies that the response to one item is conditional on the response to another item, and trait dependence suggests that another trait affects the responses [24]. LD can be identified by examining the relative correlations between standardized item residuals [24]. In the current analysis the critical value for relative residual correlations indicating LD was 0.2 [26]. If the total score consists of different domains, some LD will be expected between items in the same domain [27]. To absorb potential LD (e.g., due to domains), it is possible to merge items within each domain into a subtest to create larger polytomous items, often referred to as “testlets” [27,28]. This can be done based on an a-priori conceptual grouping (e.g., item groups according to the domains of the PCCoc conceptual framework) [27]. Signs of LD are indicated if the reliability estimate from the testlet based analysis drops considerably compared to the estimate based on the original items [24]. In addition, the testlets specific coefficients *A*, *c* and *r* are examined, where *A* is the variance common to all testlets, *c* is the variance that is unique to the testlets and *r* is the correlation between testlets corrected for attenuation due to measurement error [29]. If the scale is approximate unidimensional, a subtest will exhibit high values for *A* and *r*, and a low value for *c* [27,30].

### 2.3 Item hierarchy

Based on the PCCoc conceptual framework (Fig. 1) and discussions within the research team, 35 of the PCCoc items were ranked a-priori from lower to higher levels of person centredness (0 = personalisation; 1 = personalisation/shared decision-making; 2 = shared decision-making; 3 = shared decision-making/empowerment; 4 = empowerment). Correspondence between the empirical and conceptually expected item hierarchies was assessed using the polyserial correlation between RMM derived item locations and their a-priori expected rank order. In case of any obviously misfitting items (based on fit-statistics and ICCs), these were removed, and the analyses were repeated with misfitting items excluded. The item that exclusively represented social environment (item 1, *Inviting care environment*), was excluded prior to any analyses on theoretical basis as it is not considered to be part of the hierarchy of the conceptual framework.

All RMM analyses were conducted using the RUMM2030Plus software, version 5.8.1 (RUMM Laboratory Pty Ltd). P-values (two-tailed) <0.05 were considered significant following Bonferroni adjustment [31]. Additional analyses were carried out using R 4.3.0 (“polycor” package) and Microsoft Excel (version 2208 for Microsoft 365).

## 3 Results

There was no DIF between the two timepoints of data collection, and data from both timepoints were therefore pooled in the analyses. Six observations were excluded due to severe person misfit (i.e., fit residuals >4) (Fig. 2).

**Fig. 3:**
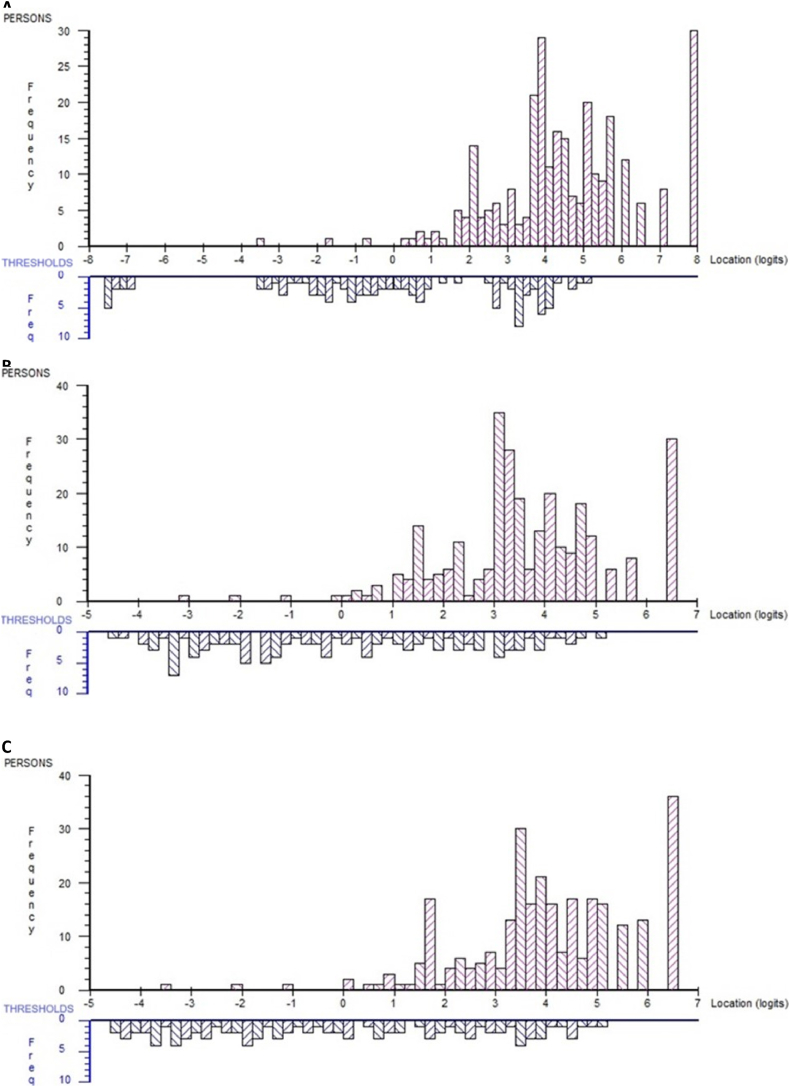
Distribution of person locations (upper red bars) and item response category threshold locations (lower blue bars) on the common logit metric (x-axis). Panel A, all 35 items; Panel B, testlets with all 35 items; Panel C, testlets with items 25 and 32 deleted.

The person-item distribution of the PCCoc displayed that most item thresholds were located between −3.5 and 5 logits (Fig. 3A). Most persons were located towards the upper end of the continuum, representing higher degrees of PCC, which is reflected by a mean (SD) person location of 4.52 (1.87).

**Fig. 4:**
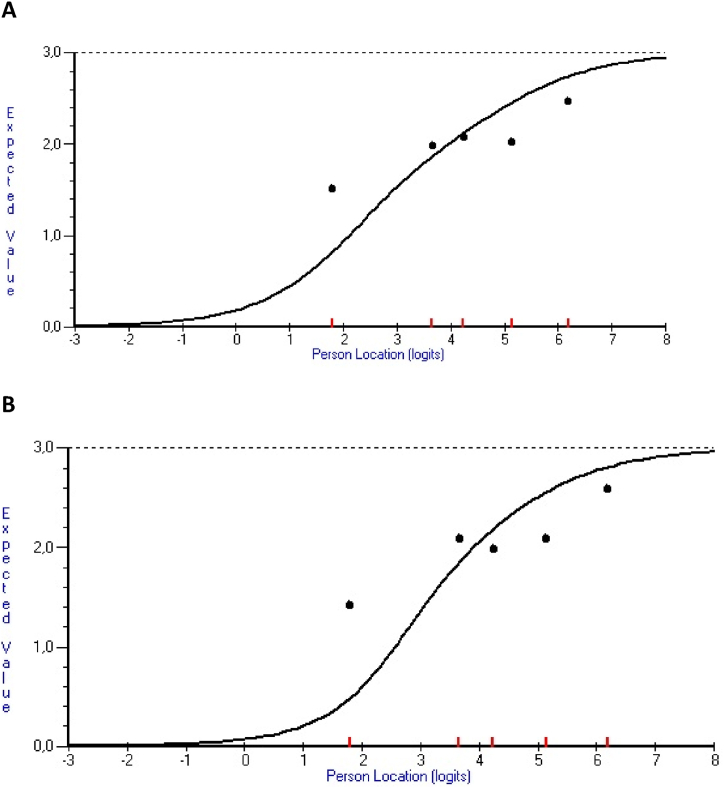
Item characteristic curves (ICCs) for items 25 (Panel A) and 32 (Panel B) with fit-residuals >2.5 and significant chi-square statistics. Ogives (ICCs) illustrate expected item responses (y-axis) for each person location (x-axis) on the latent PCC variable (positive values = higher levels of perceived PCC). Black dots denote observed item responses from groups (class intervals) of persons at comparable locations on the PCC variable (x-axis).

Individual item statistics are reported in Table 1. Two items, 25 (*Support for family members*) and 32 (*Agreed written care plan*), had fit residuals >2.5 and significant chi-square values that were considerably higher compared to the other items, and clear signs of misfit according to their respective ICC (Fig. 4A and B).

**Table 1:** Item locations, fit statistics and domains for all 35 PCCoc items ordered by location from lower to higher levels of person-centred outpatient care.

No.	Item	Location	SE	Fit residual^a^	χ^2^	P value^b^	Domain^c^
16	Care follow-up and documentation	−2.287	0.168	−1.827	14.944	0.005	P/SDM
20	Good HCP collaboration	−1.91	0.161	−1.485	6.393	0.172	SDM
4	Confirmed as person	−1.884	0.168	−1.158	6.399	0.171	P
23	Personal information documented	−1.844	0.152	−0.654	3.012	0.556	P
9	Problems are taken seriously	−1.711	0.153	−2.1	11.002	0.027	P
7	Experiences are respected	−1.679	0.149	−0.667	4.537	0.338	P
8	Self-knowledge is considered	−1.596	0.149	−2.36	6.361	0.174	P/SDM
2	Undisturbed conversations	−1.249	0.142	−1.239	12.079	0.017	P
27	Encouraged to participate	−1.157	0.136	**−2.561**	8.627	0.071	SDM
31	Participate in implementing care	−1.144	0.136	−1.417	2.993	0.559	SDM/E
29	Participate in care planning	−1.069	0.135	−1.584	3.358	0.5	SDM
18	Confident HCP contacts	−0.58	0.154	−1.499	6.853	0.144	P/SDM
5	Opportunity to tell my story	−0.267	0.155	−1.203	3.98	0.409	P/SDM
6	Understanding my situation	−0.205	0.147	−1.72	2.798	0.592	P/SDM
28	Involved in care	−0.178	0.146	**−3.113**	14.98	0.005	SDM
11	Agree with HCP on what to do	−0.103	0.148	−0.793	1.503	0.826	SDM
19	Sufficient time allocated	−0.008	0.144	0.015	1.161	0.885	P
36	Own wishes are considered	0.058	0.139	**−2.868**	7.685	0.104	SDM
24	Care information shared as needed	0.228	0.144	−0.453	1.817	0.769	SDM
26	Active participation in care	0.292	0.149	−0.896	4.843	0.304	SDM/E
10	Needs determine care planning	0.314	0.136	**−2.976**	9.785	0.044	SDM
17	Care responsibility is clear	0.377	0.132	−0.364	4.706	0.319	SDM/E
30	Participate in decisions on care	0.421	0.129	−1.151	3.37	0.498	SDM/E
3	Equality in meeting	0.541	0.137	−0.998	2.663	0.616	SDM
21	Information facilitating decisions	0.724	0.135	−1.825	5.431	0.246	SDM/E
35	Own resources are utilized	0.94	0.131	**−2.952**	11.982	0.017	SDM/E
22	Can influence care	0.941	0.131	−0.277	1.878	0.758	SDM/E
14	Coordinated care	1.07	0.128	−0.771	1.901	0.754	SDM
34	Support to achieve care goals	1.117	0.135	**−3.053**	9.66	0.047	E
33	Achieve care goals	1.176	0.133	0.296	1.992	0.737	E
12	Gain new knowledge	1.271	0.126	**3.016**	14.877	0.005	E
13	Strengthened ability to cope	1.379	0.133	−0.26	3.888	0.421	E
15	Family participation	1.55	0.121	2.387	24.089	**<0.001**	P/SDM
25	Support for family members	3.125	0.1	**8.423**	79.893	**<0.001**	SDM/E
32	Agreed written care plan	3.346	0.093	**4.989**	107.789	**<0.001**	SDM

Abbreviations: PCCoc = the Person-Centred Care instrument for outpatient care; SE=Standard error; P=Personalisation; SDM=Shared decision making; E = Empowerment.

a Values in bold indicate items with fit residuals outside the recommended ±2.5 range.

b Values in bold indicate statistical significance at the 0.05 level following Bonferroni adjustment.

c Domain(s) of the PCCoc conceptual framework that items were hypothesized to represent.

There was no DIF by gender but uniform DIF by age for items 2 (*undisturbed conversations*) and 13 (*strengthened ability to cope*). In both instances, older persons scored systematically higher than younger persons.

Response categories worked as intended with all items except for items 26 (*Active participation in care*) and 32 (*Agreed written care plan*). For these two items, response category 1 (*Disagree*) was never the most likely outcome. However, the disordering of these thresholds was within the margins of measurement error (data not shown).

### 3.1 Local dependence

There were scattered relative residual correlations above 0.2, exhibiting broad correspondence with the hypothesized hierarchical domains of the conceptual framework. Two subtest analyses were conducted to address LD based on the a-priori grouping of items into the five hierarchical domains of the conceptual PCCoc framework. First with all 35 items, and second with misfitting items (items 25 and 32) excluded. Both analyses showed improved model fit (Table 2), a more even threshold distribution without any major gaps (Fig. 3B and C) and no DIF. In addition, both subtest analyses supported the unidimensionality of the PCCoc (Table 2).

**Table 2:** Overall scale statistics from individual item-level and testlet based analyses of the PCCoc.

	Item-level analysis 1^a^		Testlet analysis 1^b^		Item-level analysis 2^c^		Testlet analysis 2^d^	
		p-value		p-value		p-value		p-value
Item-trait interaction^e^, χ^2^	409.2	<0.001	24.8	0.21	226.4	<0.001	28.9	0.09
PSI^f^	0.91		0.9		0.91		0.89	
Strata^g^	4.8				4.5			
α^h^	0.96		0.92		0.97		0.93	
α diff.^i^			−0.04				−0.04	
*A*^j^			0.96				0.97	
*c*^k^			0.25				0.25	
*r*^l^			0.94				0.94	

a Analysis based on 35 PCCoc items.

b Testlet analysis of 35 PCCoc items conceptually grouped according to their assumed representation of five hierarchical domains.

c Analysis based on 33 PCCoc items (item 25 and item 32 excluded).

d Testlet analysis of 33 PCCoc items (items 25 and 32 excluded) conceptually grouped according to their assumed representation of five hierarchical domains.

e Should be nonsignificant to support overall model fit.

f Person separation index.

g Number of statistically distinct groups of persons that can be distinguished by the instrument.

h Coefficient alpha.

i Difference between α from item-level and testlet based analyses.

j Variance common to all testlets.

k Variance that is unique to the testlets.

l Correlation between testlets.

### 3.2 Item hierarchy

**Fig. 5:**
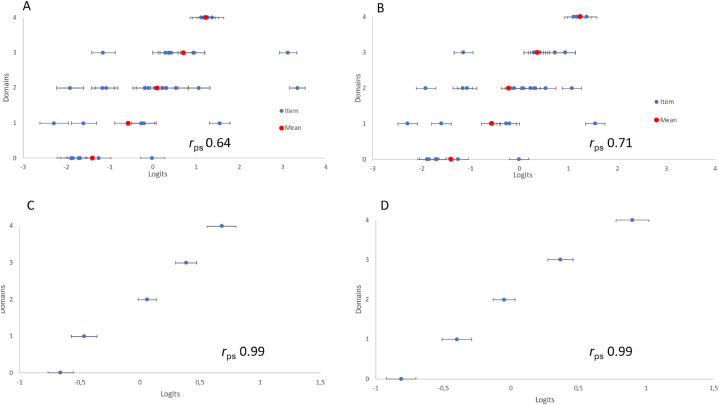
Individual item and testlet hierarchies of the PCCoc. Based on the conceptual framework, 35 of the PCCoc items were ranked a-priori in 5 domains according to the conceptual framework, representing less to more person centredness (0 = personalisation 1 = personalisation/shared decision-making 2 = shared decision-making 3 = shared decision-making/empowerment 4 = empowerment). Panel A (all 35 items) and Panel B (items 25 and 32 excluded) displays the item hierarchy according to their location on the linear logit (log-odd units) scale (x-axis) and the a-priori ranked domains (y-axis). Panel C (all 35 items) and Panel D (items 25 and 32 excluded) displays the testlet hierarchy with all items a-priori identified in each of the 5 domains merged into 5 corresponding testlets. *r*_ps_ = the polyserial correlation between the item locations on the logit scale and their rank order.

Table 1 shows the PCCoc items ordered by their locations on the hierarchical continuum from less to more PCC, as well as the respective item's a-priori hypothesized domain, providing an overview of the empirical distribution of the overlapping domains along the hierarchical continuum. Fig. 5 shows the individual item localisations and the mean item locations within each domain. As can be seen, locations generally follow the conceptual hierarchy (Fig. 5A and B). The polyserial correlation (95 % CI) between empirical item locations and the expected rank order using all 35 PCCoc items was 0.64 (0.44–0.84). After exclusion of two obviously misfitting items (items 25 and 32; see above), the correlation (95 % CI) was 0.71 (0.55–0.87). Testlet locations using all 35 PCCoc items as well as with misfitting items excluded, showed closer correspondence with the conceptual hierarchy (Fig. 5C and D).

To explore the potential influence of combining data from the two timepoints, the above analyses were repeated with data from only the first timepoint. This showed similar results with a polyserial correlation of 0.6 for all 35 items (0.65 with items 25 and 32 excluded) and 0.99 for testlet locations (with and without items 25 and 32).

## 4 Discussion

The aim of this study was to investigate how empirical PCCoc item response data from persons with LTCs attending different outpatient clinics relate to the conceptual hierarchal framework from lower to higher levels of perceived person-centred outpatient care.

Two findings indicate a correspondence between empirical PCCoc data and the conceptual hierarchy from lower (personalisation) to higher (empowerment) levels of perceived person-centred outpatient care. Firstly, empirical item locations correlated with the hierarchically a-priori ranked items according to the conceptual framework. This finding was also supported graphically. Secondly, testlets of hierarchically grouped items exhibited a strong correlation and a clear graphical correspondence between their locations and the expected hierarchy based on the conceptual framework. This is important since a fundamental assumption of the RMM is that the item order from lower to higher reflects differences in the amount of the underlying latent variable [10], which calls for a clear definition of the underlying framework on which the instrument is based [8,9,11]. However, there seems to be a deficiency of PREMs for outpatient care that have been tested based on modern test theory, such as RMT [6]. Furthermore, to the best of our knowledge, there is a scarcity of instruments with the intention of measuring perceived PCC in outpatient care that are based on a conceptual framework that articulates how the target variable varies from less to more.

As expected, residual correlations suggested the presence of LD. Testlets were used to absorb LD [27,28], and a comparison between item level and testlet level analyses was conducted to gain a refined understanding of the impact of LD [25]. The decrease in coefficient alpha was small, indicating a small impact of the identified LD [25]. Furthermore, the variance common to all testlets (the *A* coefficient) in the analysis was high whereas the variance unique to the testlets (the *c* coefficient) was low, and the correlation between testlets (the *r* coefficient) was high. Taken together, this implies that the PCCoc can be considered unidimensional [27,29], and that the observed LD is best explained by expected correlations between items within the respective hierarchical domains of the PCCoc conceptual framework. Dimensionality has been described as a relative issue [32], and PCC itself has been described as having multiple dimensions [1,33] and to be context-specific [33]. This means that a unidimensional variable does not exist *per se* but needs to be constructed in order to make comparisons based on differences in degree [32], e.g., differences in perceived PCC. A consequence of this is the need for a clearly defined framework of how the latent PCC variable is to be understood in the specific context in which the quantification is of interest. Our observations suggest that the hierarchical PCCoc framework may represent such a framework for person-centred outpatient care.

In general, the PCCoc seems to fit the RMM, and reliability estimates were all well above the recommended minimum of 0.70–0.80 [19,22], suggesting that the instrument is able to separate persons into 4–5 statistically distinct strata. Two items (25 and 32) showed clear signs of deviation from the RMM. This is not surprising since both these items have been flagged for revision based on qualitative patient input [12]. The PCCoc has subsequently been revised with the aim of addressing identified ambiguities with these items. However, it can be noted that other instruments have also described difficulties with similar items [34,35].

## 5 Limitations

The sample size can be considered somewhat limited, which leads to less precise estimates and larger standard errors [17,36]. Sample sizes between n = 250 to n = 500 have been suggested reasonable regarding interpretation of fit-statistics [37,38] but may need to be adjusted upwards depending on the number of thresholds [36]. There is a potential limitation in combining data from two timepoints. While independent data from one timepoint is more ideal, merging of timepoints enables a larger sample and improved precision of the estimates. Separate analyses using data from only one timepoint indicated that merging data did not affect the results in any meaningful way.

Due to the small sample from each outpatient clinic, it was not meaningful to examine DIF by specialty. The response categories in the PCCoc appeared to work as intended, but there were few or no responses in some categories, which increases the uncertainty in threshold estimations. The targeting of the PCCoc was skewed, where the PCCoc failed to capture higher degrees of perceived PCC and there were few persons experiencing lower degrees of PCC. However, this appears to be general feature of this type of instruments [34,35,39,40].

There is a need for further studies with larger samples for a more thorough psychometric examination of the PCCoc, e.g., threshold ordering as well as any considerations regarding removal of items. The study also includes a relatively limited number of groups within LTCs, and additional groups may be included in the future for a more comprehensive representation of this heterogeneous target group. However, the purpose of the present study was to test, at a relatively early stage in the development of the PCCoc, whether empirical data from different outpatient clinics support the hierarchy of the conceptual framework, as an important step in a rigorous instrument development process [8,10,11].

## 6 Conclusions

This study provides evidence that empirical PCCoc data correlate with its underpinning conceptual framework of person-centred outpatient care, and that the PCCoc appears to be a unidimensional PREM. This means that the PCCoc may support healthcare providers in evaluating the degree to which persons with LTCs perceive their care to be person-centred and guide the providers to well-targeted interventions for a more person-centred care.

## CRediT authorship contribution statement

**Fredrik Gasser:** Writing – original draft, Methodology, Formal analysis, Conceptualization. **Albert Westergren:** Writing – review & editing, Methodology, Conceptualization. **Sidona-Valentina Bala:** Writing – review & editing, Methodology, Conceptualization. **Joakim Ekstrand:** Writing – review & editing, Methodology, Conceptualization. **Peter Hagell:** Writing – review & editing, Methodology, Formal analysis, Conceptualization.

## Consent to participate

Written informed consent was obtained from all study participants.

## Data availability

The datasets generated and/or analysed during the current study are available from the corresponding author on reasonable request.

## Ethics approval

The study was approved by the Swedish Ethical Review Authority (Dnr. 2021-00620; approval date February 22, 2021)

## Funding

This study was funded by 10.13039/501100008408Kristianstad University.

## Declaration of competing interest

The authors declare that they have no known competing financial interests or personal relationships that could have appeared to influence the work reported in this paper.
